# Mechanomyography and acceleration show interlimb asymmetries in Parkinson patients without tremor compared to controls during a unilateral motor task

**DOI:** 10.1038/s41598-021-81672-z

**Published:** 2021-01-29

**Authors:** Laura V. Schaefer, Nils Löffler, Julia Klein, Frank N. Bittmann

**Affiliations:** Division Regulative Physiology and Prevention, Department Sports and Health Sciences, University of Potsdam, Karl-Liebknecht-Str. 24-25, house 24, 14476 Potsdam, Golm, Germany

**Keywords:** Neuroscience, Diseases, Neurology

## Abstract

The mechanical muscular oscillations are rarely the objective of investigations regarding the identification of a biomarker for Parkinson’s disease (PD). Therefore, the aim of this study was to investigate whether or not this specific motor output differs between PD patients and controls. The novelty is that patients without tremor are investigated performing a unilateral isometric motor task. The force of armflexors and the forearm acceleration (ACC) were recorded as well as the mechanomyography of the biceps brachii (MMGbi), brachioradialis (MMGbra) and pectoralis major (MMGpect) muscles using a piezoelectric-sensor-based system during a unilateral motor task at 70% of the MVIC. The frequency, a power-frequency-ratio, the amplitude variation, the slope of amplitudes and their interlimb asymmetries were analysed. The results indicate that the oscillatory behavior of muscular output in PD without tremor deviates from controls in some parameters: Significant differences appeared for the power-frequency-ratio (*p* = 0.001, *r* = 0.43) and for the amplitude variation (*p* = 0.003, *r* = 0.34) of MMGpect. The interlimb asymmetries differed significantly concerning the power-frequency-ratio of MMGbi (*p* = 0.013, *r* = 0.42) and MMGbra (*p* = 0.048, *r* = 0.39) as well as regarding the mean frequency (*p* = 0.004, *r* = 0.48) and amplitude variation of MMGpect (*p* = 0.033, *r* = 0.37). The mean (M) and variation coefficient (CV) of slope of ACC differed significantly (M: *p* = 0.022, *r* = 0.33; CV: *p* = 0.004, *r* = 0.43). All other parameters showed no significant differences between PD and controls. It remains open, if this altered mechanical muscular output is reproducible and specific for PD.

## Introduction

There is plenty of research dealing with the investigation of pathomechanisms or the search for a possible biomarker for Parkinson’s disease (PD). Currently, the diagnosis is based on the UK Parkinson’s Disease Society Brain Bank Diagnostic Criteria, which especially covers clinical examinations using the Unified Parkinson’s Disease Rating Scale (UPDRS)^1–4^. Clinical examinations are critisised to be subjective, although some investigations found the UPDRS to be at least sufficient reliable^5–9^. The clinical findings can be supplemented by various additional examinations (SPECT, midbrain sonography, odor test, polysomnography). Several researchers point out the need for an objective diagnostic tool for PD^10–12^, which can be quickly and easily applied in addition to the routine procedures. Potential biomarkers for diagnosis of PD, which are currently investigated, are presented in Kalia and Lang^10^. A biomarker using the motor output is not considered therein. In a recent paper, Evers et al.^11^ pointed out to record sensor-based parameters of motor output as gait parameters and tremor-related items to monitor PD. However, not each PD patient exhibits a tremor, especially not in the early stages. A clinical diagnosis in prodromal stages is difficult, since the cardinal symptoms rigor, resting tremor and bradykinesis are often extremely discreet. A challenge arises from the task to identify persons at risk even prior to the manifestation of the cardinal symptoms (premotor stage). If the motor stage is reached, a significant degeneration of at least 50% decline of the dopaminergic neurons has already occured^13,14^. Therefore, a main objective is to identify an early diagnosis to probably prevent further loss through an early started intervention^15^.

Kalia & Lang^10^ stated that “Parkinson’s disease is now viewed as a slowly progressive neurodegenerative disorder that begins years before diagnosis can be made” (p. 896). Therefore, it is assumed that discreet changes in motor output might exist in the preclinical phase. According to McAuley and Marsden^16^ the mechanical muscle oscillations might serve as a “’window’ into the function of central oscillations”. They pointed out the obvious limitations to directly record the central oscillations. So, why not use the motor output to provide information of central abnormalities? Several researchers examined gait parameters as stride duration, arm and leg swing as well as step time in PD, which partly showed changes already in prodromal stages^17–23^. Deviation in those parameters seem not to be specific for PD, since they do not only occur in PD, but also in diseases like Alzheimer’s or Huntington’s diesaese^23–25^.

A further parameter to monitor the motor output is the mechanomyography (MMG), which is used to record the muscular micro-oscillations. The research group around Marusiak et al.^ 26,27^ investigated MMG in PD patients with tremor. However, in PD patients with apparent tremor, the detection via MMG or a common tremor measurement is not necessary because a visual diagnosis can be performed easily. Of course, it can provide information about the characteristics of the tremor; however, it is not of great value for diagnosis. The muscular micro-oscillations might probably show deviations already before the tremor is apparent. Therefore, the objective of this study was to investigate, whether or not Parkinson's patients without a clinical tremor show altered oscillation patterns in the mechanical muscular output.

## Methods

The aim of this exploratory study was to examine how the mechanical myofascial oscillations behave during a unilateral isometric motor task in patients with Parkinson’s disease without tremor compared to healthy controls. A similar investigation on the same population was already done in a specific bilateral isometric motor task, whereby the subject was interacting with itself^28^. Therefore, some parts of this methods section are overlapping with the methods in Schaefer & Bittmann^28^.

## Participants

### Controls

There were 29 healthy subjects who volunteered to participate in the study. They were recruited from the Club Aktiv of the Brandenburgischer Verein für Gesundheitsförderung e.V. (BVfG; Brandenburg Association of Health Promotion; Potsdam, Germany) or from relatives of the included patients. Exclusion criteria were complaints of the upper extremities, the shoulder girdle and cervical spine within the last six month, a malignant hypertension and any hints for a neurological disease. The healthy controls passed a neurological examination performed by neurologists of the Neurological Clinic for Movement Disorders and Parkinson’s Disease (Beelitz-Heilstätten, Germany). Four participants had to be excluded because of the results of that examination. The anthropometric data and the averaged maximal voluntary isometric contraction (MVIC) during the isometric tasks for the armflexors of the remaining 25 healthy subjects (*n* = 12 male, *n* = 13 female) are displayed in Table 1. Two subjects were left-handed. The remaining 23 controls were right-handed^28^.

**Table 1:** Anthropometric data and averaged values of the maximal voluntary isometric contraction (MVIC) (arithmetic mean ± standard deviation (SD)) of all included subjects.

Gender	PD-patients		Controls	
	m	f	m	f
n	18	4	12	13
age [years]	62.88 ± 10.36	66.75 ± 9.81	69.83 ± 6.00	67.23 ± 7.55
BMI	27.82 ± 4.40	26.64 ± 7.78	25.29 ± 2.62	25.97 ± 3.39
MVIC left [Nm]	49.85 ± 20.09	28.10 ± 8.66	62.58 ± 19.40	32.96 ± 9.63
MVIC right [Nm]	49.80 ± 16.61	28.11 ± 9.60	65.26 ± 21.75	33.93 ± 10.06

### Patients with Parkinson’s disease

There were 28 patients diagnosed with Parkinson’s disease (PD) *without a tremor* who volunteered to be part of the PD group. The patients were recruited from the Neurological Clinic for Movement Disorders and Parkinson’s Disease in Beelitz-Heilstätten (Germany; Chief physician: Prof. Dr. G. Ebersbach) and were measured in the clinical routine during the medical off phase (~ 12 h after medication intake). Exclusion criteria included the appearance of a clinical tremor, neurological symptoms beyond PD, a manifest polyneuropathy, a malignant hypertension, a coronary heart disease of NYHA III or higher, brain pacemaker, brain aneurysms, glaucoma and hemorrhagic apoplex. Relative exclusion criteria were orthopedic symptoms of the upper extremities, the shoulder girdle and cervical spine within the last six months.

In total, six patients had to be excluded due to their medical history (*n* = 4) or signal quality (*n* = 2). The anthropometric data and the MVIC of the armflexors are displayed in Table 1^28^.

### Setting

**Figure 1:**
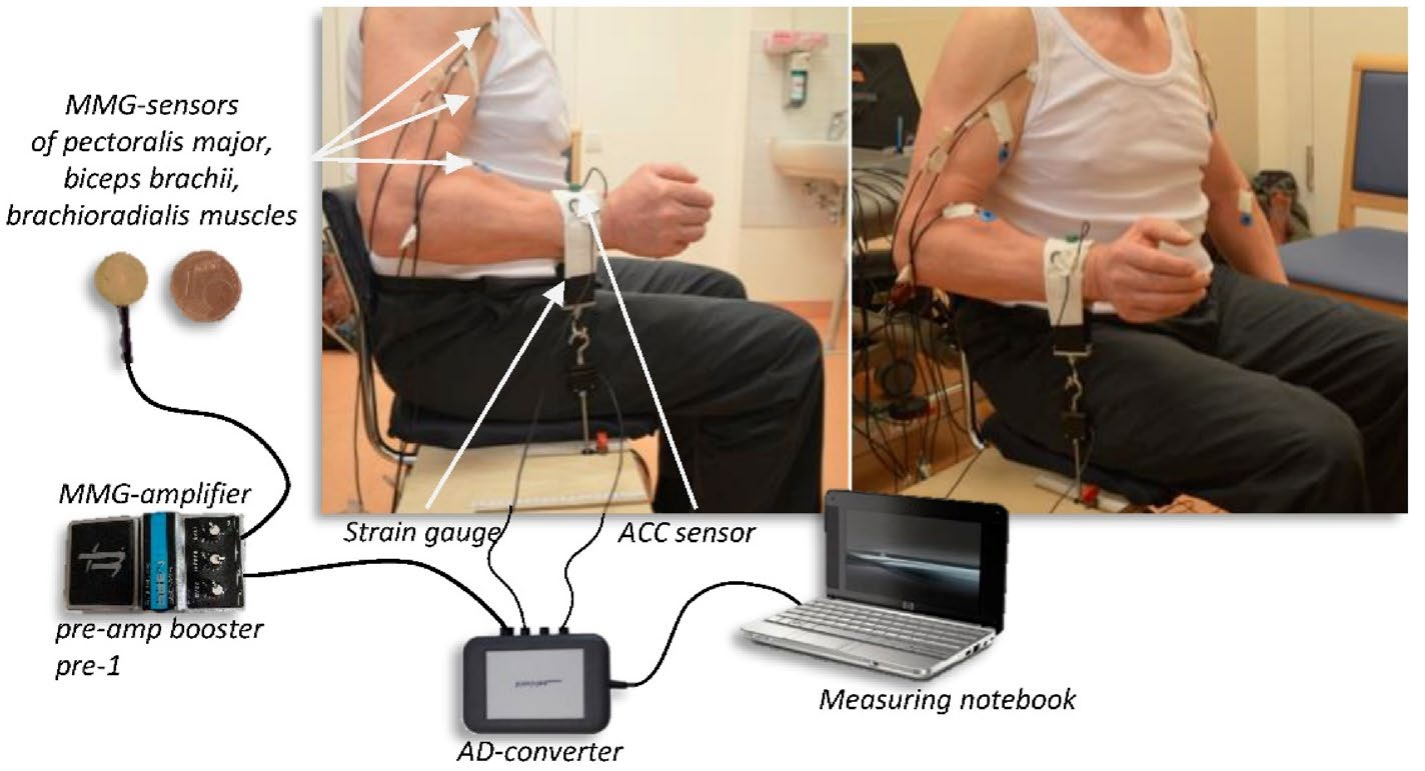
Setting for the unilateral isometric motor task. The MMG-sensors were fixed above the biceps brachii, brachioradialis and pectoralis major pars clavicularis muscles and amplified using the Nobels pre-amp booster pre-1. The strain gauge, which was fixed by a strap to the forearm of the participants, recorded the force and the ACC-sensors captured the motion and accelerations of the forearm. All signals were transmitted through an AD-converter to the measuring notebook.

In the setting-up (Fig. 1) the subjects sat on a specially customised chair with 90° hip and knee angle. A plate was attached to the chair to fix the strain gauge either on the left or on the right side of the subject with the possibility to adjust the position of the strain gauge in the depth. The strain gauge (model: ML MZ 2000 N 78, 2000 N, modified by biovision) was fixed in a rail of the plate and was connected to a strap. The strap enclosed the distal forearm of the participant, so that the subject was able to pull on the strain gauge. Thereby, the arm was adducted with 90° flexion in the elbow joint, the hand was in neutral position. The acceleration sensor with a sensitivity of 312 mV/g (range ± 2 g, linearity: ± 0.2%; comp. biovision) was fixed on the strap to detect the accelerations along the longitudinal acting force vector. The mechanical oscillations of the biceps brachii (MMGbi), the brachioradialis (MMGbra) and of the pectoralis major muscles (MMGpect) were recorded using piezoelectric sensors (Shadow SH 4001). They were fixed on the skin above the muscle bellies with ECG-tape. The MMG-signals were conducted across an amplifier (Nobels preamp booster pre-1). All signals were converted by an ADC (National Instruments, 14-bit, USB 6009; modified by Biovision) and subsequently were recorded by the software NI DIAdem 2012 (National Instruments) on a measurement notebook (Toshiba Satellite Pro L500-1T2; Windows 7). Sampling rate was set at 1000 Hz^28^.

### Measuring procedure

Prior to measurements, the patients were examined by various neurologists using the UPDRS. For organisational reasons, the controls were examined neurologically in an extra appointment. Afterwards, the subject was introduced to the system and procedure. The subject was then seated on the chair and was equipped with the sensors. The unilateral isometric task (in total 10 trials) was performed randomized (coin toss) for the left and right side: The first trial was performed in a resting position to indicate a potential resting tremor. Thereby, the subject had its hands on lap. The second trial was performed in the starting position (without pulling on the strap) to detect a potential tremor. Afterwards, the MVIC was identified in two trials, in which the subject had to pull maximally on the strap. The higher of the two maximal values was then used to calculate 70% of the MVIC for the further five trials. Thereby, the subject had to pull on the strap until 70% of the MVIC was reached and maintain this force level for 8 s. The intensitiy could be controlled via biofeedback on a screen (pointer display). The resting period between the trials of MVIC and of 70% of the MVIC was set at 90 s. The last trial was done in the resting position again to once more determine a possible tremor. Subsequently, the same procedure was performed with the contralateral side.

### Data processing and statistical analysis

The software NI DIAdem 14 was utilised for the data processing and partly for the analysis. Excel (Microsoft Office, 2013) was used for further processing and SPSS Statistics 25 (IBM) for statistical analyses. The isometric plateau at 70% of the MVIC was cut from the raw data of each trial (reference: force signal; deviations of ± 10% were accepted) and was used for further analyses. Various parameters were taken into account: (1) MVIC (force signal); and the following parameters concerning the oscillating signals of MMGs and ACC within one trial: (2) a specific ratio (Q_REL_) of the Power Spectral Density (PSD); (3) the slope of the amplitude maxima; (4) the variation of the amplitude maxima and (5) the mean frequency^28^.

For the analysis of oscillating signals, the raw signals must have a signal-to-noise-ratio (SNR) of at least 10 dB^29^. Signals with a lower SNR were excluded (Table 2). This is the reason why some comparisons are based on lower sample sizes. The following explanations concerning the five parameters are based on the description in Schaefer & Bittmann^28^.

**Table 2:** Included signals. Included signals after considering the necessary signal to noise ratio (SNR) of > 10 dB of raw signals differentiated by sensor and side.

	PD		Controls	
	Left	Right	Left	Right
ACC	12	10	17	9
MMGbi	18	19	23	22
MMGbra	14	15	14	18
MMGpect	18	15	21	21

1. *Maximal voluntary isometric contraction (MVIC)*

The maximum value of the filtered force signal (filter type: Butterworth; filtering degree 10, cutoff frequency 3) was determined in NI DIAdem. The higher value of both MVIC trials was transmitted in SPSS for group comparisons^28^.

(2) *Specific power ratio Q*_*REL*_

The idea behind this parameter is to get the percentage of the power in the low frequency range of 3 to 7 Hz (Interval 1; I1) on the power in the frequency range of 3 to 12 Hz (Interval 2; I2). This was compiled since in descriptive analysis two peaks were identified in PSD of PD patients: one in a lower and one in a higher frequency range. By calculating the mean frequency those peaks would be eliminated^28^.

**Figure 2:**
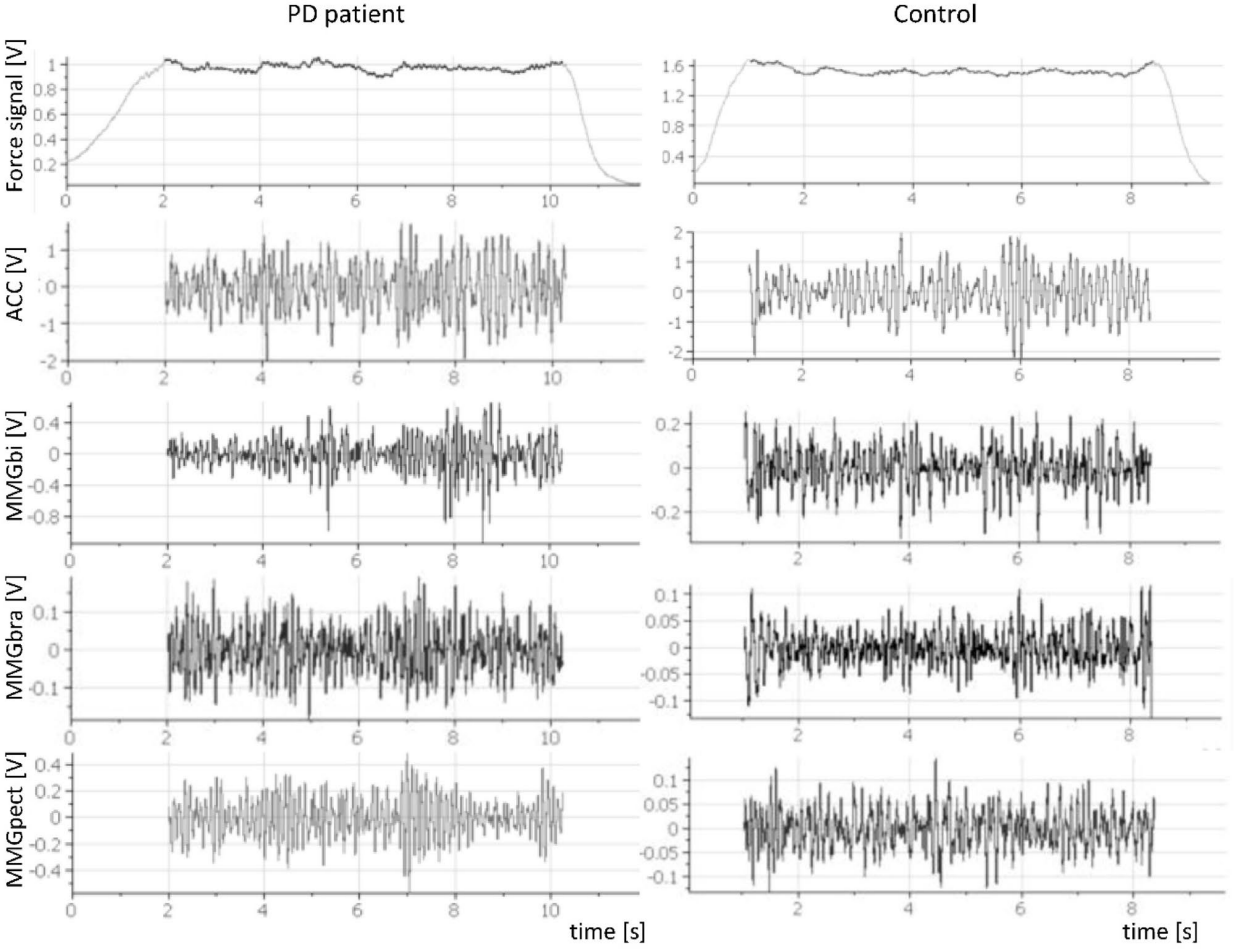
Exemplary raw signals of one PD patient and one control. The left panels display the raw signals of force, ACC, MMGbi, MMGbra, MMGpect of the left side of a PD patient during one trial of the unilateral task at 70% of the MVIC, the right panels illustrate the same signals of one participant of the control group. The dark area in the force signals indicates the isometric plateau.

The raw data (Fig. 2) were used to estimate the PSD, which is the basis for calculating Q_REL_:

$$ \begin{aligned} Q_{REL} = \frac{{{\text{M of power in the frequency range of }}3{\text{ to 7 Hz}}}}{{({\text{M of power in frequency range of }}3{\text{ to 7 Hz) }} + ({\text{M of power in frequency range of }}7{\text{ to }}12{\text{ Hz)}}}}  \end{aligned}$$

For each trial and each MMG-/ACC-signal one value Q_REL_ results. For further analysis the following variables were calculated out of the five power ratios Q_REL_:^28^

I. Arithmetic mean (M) of the five Q_REL_ for each signal (MMG, ACC) and side:
  1. **MQ**_**REL**_ = absolute value of MQ_REL_
  2. Relative asymmetrie [percentage points (pp)] of left (le) and right (ri) side of MQ_REL_:
    $${\mathbf{Asym}}-{\mathbf{MQ}}_{{{\mathbf{REL}}}} = \left| {\left( {\frac{{MQ_{REL} le}}{{MQ_{REL} le + MQ_{REL} ri}} \cdot 100} \right) - 50} \right|$$
II. Coefficient of variation (CV) of the five Q_REL_ for each signal (MMG, ACC) and side:
  1. **CVQ**_**RE**L_ = absolute value of the CV
  2. **Asym-CVQ**_**REL**_ = relative asymmetrie [pp] of left and right side of CVQ_REL_: (Analogue to I.2).

### Data processing for parameters (3) to (5)

The MMG and ACC-signals (isometric plateau) were filtered (Butterworth, filtering degree 5, cutoff frequency 20 Hz) and the maxima of each amplitude were determined^28^.

(3) Slope of the amplitude maxima

To calculate the mean slope of all maxima of one signal, the slope function in Excel was used. In order to create a linear parameter for further consideration, this was converted into degree. One slope value resulted per trial for each signal and side^28^.

(4) Amplitude variation (VAmp)

For the variation of amplitude within one trial, the absolute difference between the y-values of two consecutive maxima was calculated in Excel. The resulting differences were averaged per trial and were relativized to the arithmetic mean of the amplitudes. One value VAmp resulted per trial for each signal and side^28^.

(5) Mean frequency

The frequency of one signal was calculated by, firstly, determining the reciprocal of the time distances (x-values) between two consecutive maximum data points and, secondly, averaging these values of one signal. This method is rather unusual for stochastically distributed variables as captured here. However, concerning Pikovsky et al.^30^ it is possible to use the reciprocal of period duration in almost periodic oscillations in chaotic systems, as the neuromuscular system. The authors regard this technique as appropriate for the present investigation to get information about the mean frequency, considering that, thereby, the amplitudes are not taken into account. The latter is done by parameter (2)^28^.

For further statistical analyses of the parameters (3), (4) and (5), the arithmetic mean (M) and coefficient of variation (CV) were calculated for all signals for the left and right side using the values of the five trials. Furthermore, the relative asymmetrie (Asym) of the left and the right side was calculated (interlimb-asymmetry). Hereby, the sides of PD patients were divided into ‘not or less affected’ and ‘more affected’ on the base of the results of the UPDRS^28^.

### UPDRS

The scores of the UPDRS concerning motor control (items 18–31) were estimated by the neurologists. A maximal amount of 108 points may be obtained thereof. A healthy person should have a value of zero. In the course of data analysis, the estimation of the more affected side using the UPDRS was compared to the own perception of the patient.

The differentiation of sides (not-less affected/more affected) are based on the UPDRS scores. A difference between UPDRS values of left and right side of more than 2 points are considered as real side difference (proposed by the neurologists). However, the analysis of interlimb-asymmetry included all participants. The consideration of the less and more affected side of PD patients within the PD group was performed additionally by including exclusively patients with an UPDRS difference of more than 2 pts.

### Statistical considerations

Each parameter in each group (PD vs. Con) was checked for normal distribution by using a Shapiro-Wilk test. For the group comparisons of anthropometric data and MVIC a one-way ANOVA was performed, including the Bonferroni post-hoc test. The gender differences were analysed using the Chi-squared test^28^.

For the parameters (2) to (5) an unpaired t-test for parametric data or a Mann-Whitney-U test for non-parametric data were utilised to compare the groups PD and Con. For ANOVA and t-test the Levène test of variance homogeneity was performed and required. If variance homogenity was not fulfilled, a Welch correction was performed. For the comparisons of less affected and more affected side of the PD, the Wilcoxon (non-parametric data) or the t-test for paired samples (parametric data) were used.

The effect size was determined either with the Pearson’s correlation coefficient (*r*) for parmetric data or with the Cohen’s *r* for non-parametric data. Furthermore, the 95% confidence intervals (CI) were calculated for the parameters (2) to (5). For correlation of UPDRS and MVIC the Spearman correlation coefficient was utilised. Significance level was set at α = 0.05^28^.

### Data availability

The datasets generated and/or analysed during the current study are available from the corresponding author on request.

### Ethical approval

The study was approved by the ethic committee of the University of Potsdam (Germany; approval no. 60/2016) and by the State Chamber of Medicine in Brandenburg (Germany). It was conducted in accordance with the declaration of Helsinki. All subjects/participants were informed in detail and gave their informed written consent to participate.

### Informed consent

Informed written consent was obtained from all individual participants included in the study. Furthermore, the participant in Fig. 1 approved to be published.

## Results

### Anthropometric data and MVIC

The anthropometric data did not differ statistically significant between the groups regarding to age (*t*(41) = − 2.045; *p* = 0.063) and BMI (*t*(41) = 1.229, *p* = 0.229). The proportion of female and male over the whole sample (PD and Con) was statistically not significant (*χ*^*2*^ = 7.739, *p* = 0.052). The amount of male participants (*n* = 17) was significantly higher than the amount of females (*n* = 4) in the PD group (*χ*^*2*^ = 8.048, *p* = 0.005) compared to the controls (*n* = 12 male, *n* = 13 female; *χ*^*2*^ = 0.040, *p* = 0.841) (Table 1).

All MVIC data were normally distributed and the variance homogeneity was fulfilled for both groups. As displayed in Table 1, the arithmetic mean of the MVIC of male controls were approximately 11 Nm (left) and 12 Nm (right), respectively, higher compared to the PD-group. Female controls showed an approximately 5 Nm (left) and 6 (right) Nm higher MVIC compared to the PD group. However, the difference of MVIC between the whole PD and control groups was not significant (left: *t*(43) = − 0.043, *p* = 0.966; right: *t*(43) = − 0.155, *p* = 0.878). The ANOVA was, as expected, found to be significant for MVIC between the gender independent of the groups control or PD (left: *F*(41,3) = 0.922, *p* = 0.000; right: *F*(41,3) = 11.219, *p* = 0.000). The post-hoc Bonferroni test displayed no significant differences between the groups PD and controls within the females or males (female left: *p*_*adj*_ = 1.0; right: *p*_*adj*_ = 1.0; male left: *p*_*adj*_ = 0.530; right: *p*_*adj*_ = 0.270). Therefore, the following analyses are based on similar force intensities between the groups PD and Con. The Spearman correlation coefficient showed no significant correlation between UPDRS score and MVIC (left: *p* = 0.287, right: *p* = 0.443).

### Specific ratio Q_REL_

**Figure 3:**
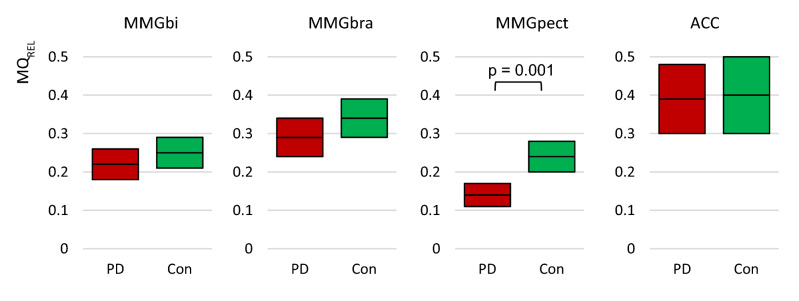
Specific ratio MQ_REL_. Displayed are the 95% confidence intervals of the arithmetic mean of the specific ratio Q_rel_ (MQ_REL_) of the MMGs and the ACC signals. As can be seen in the MMG-signals, the PD have a lower ratio compared to controls. However, only concerning the MMGpect significant differences of p = 0.001 occur. The CIs of the ACC signal are overlapping completely.

The 95% CIs of the variable MQ_REL_ for the MMG and ACC signals compared between PD and Con are displayed in Fig. 3, the related statistical parameters are given in Table 3. The MQ_REL_ of MMGbi and MMGbra differed not significantly between PD and Con (*p* > 0.05), whereas the MMGpect showed a significantly higher MQ_REL_ in Con compared to PD (*t*(58) = -3.652, *p* = 0.001, *r* = 0.432).

**Table 3:** Statistical values of parameters of Q_REL_ for each signal. Displayed are the arithmetic mean and SD, significance p of t-test for independent variables and effect size r of MQ_REL_ and CVQ_REL_ and their side asymmetries (Asym-MQ_REL_; Asym-CVQ_REL_ comparing PD and Con regarding the signals MMGbi, MMGbra, MMGpect and ACC.

	MMGbi		MMGbra		MMGpect		ACC	
	PD	Con	PD	Con	PD	Con	PD	Con
Sample size n	33	39	29	32	29	32	18	28
MQ_REL_	0.22 ± .11	0.25 ± .13	0.29 ± .14	0.34 ± .15	0.14 ± .09	0.24 ± .11	0.40 ± .21	0.39 ± .25
Sign p (effect r)	.222		.225		**.001 (.432)**		.961	
Asym-MQ_REL_ [pp]	15.9 ± 10.6	8.37 ± 6.2	12.02 ± 7.4	7.05 ± 4.4	15.9 ± 10.2	17.0 ± 10.5	11.3 ± 9.8	10.4 ± 11.0
Sign p (effect r)	**.008 (.423)**		**.048 (.391)**		.315		.867	
CVQ_REL_ [%]	33 ± 18	28 ± 14	23 ± 13	28 ± 12	38 ± 15	31 ± 18	31 ± 16	31 ± 20
Sign p (effect r)	.268		**.048 (.254)**		.171		.926	
Asym-CVQ_REL_ [pp]	14.2 ± 10.7	12.6 ± 7.7	15.8 ± 9.1	9.0 ± 7.1	7.7 ± 5.9	13.1 ± 7.1	16.6 ± 11.5	12.0 ± 10.4
Sign p	.606		**.043 (.399)**		.052		.435	

Significant results are displayed in bold.

**Figure 4:**
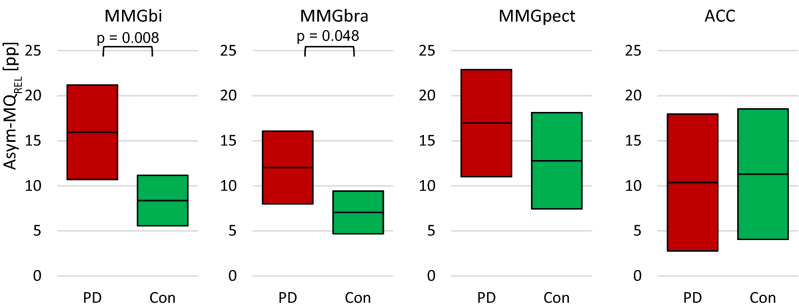
Interlimb asymmetries of ratio MQ_REL_. Displayed are the 95% confidence intervals of the side differences of the arithmetic mean of the specific ratio Q_rel_ (Asym-MQ_REL_) of the MMGs and the ACC signals. As can be seen in the MMG-signals of the elbow flexors, the PD have a higher side difference compared to the controls (p = 0.008; p = 0.048). In the MMGpect, a tendency of higher side asymmetry in PD is visible, whereas the ACC-signal show no difference between PD and Con.

**Figure 5:**
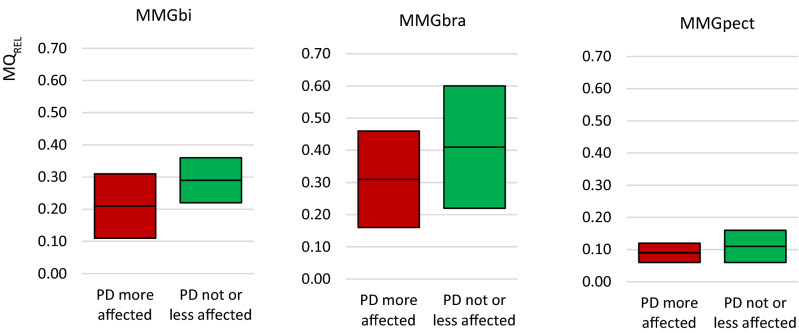
Comparison of less and more affected side in PD with UPDRS-difference > 2 pts. Displayed are the 95%-CIs of the arithmetic mean of Q_rel_ (MQ_REL_) of the MMG-signals comparing the more affected and the not or less affected side in PD. Only PD patients with a difference of > 2 pts in UPDRS were included. As can be seen, the more affected side tend to have a lower ratio compared to the less affected side. However, the data are not significant.

**Figure 6:**
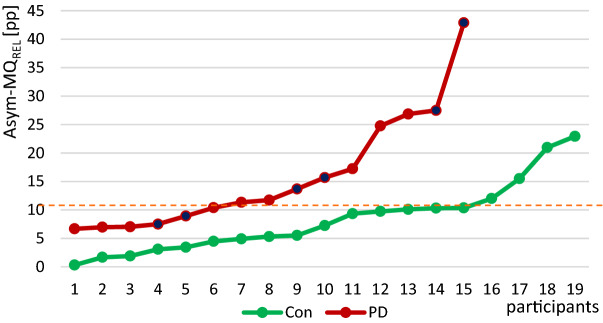
Relative interlimb asymmetry of MQ_rel_. Displayed are the relative differences between left and right MMG of biceps brachii muscle according to the parameter MQ_REL_ between the PD patients and the controls sorted by size. The dotted line shows a possible boarder according to the 95%-CI at approximately 11 pp difference. The dark filled circles represent the PD patients with a side difference in UPDRS of > 2 pts.

The relative asymmetry of MQ_REL_ (Asym-MQ_REL_[pp]) between left and right side was found to be significant for MMGbi and MMGbra between PD and Con, but not for the MMGpect (Fig. 4; MMGbi: *U* = 67.00, *p* = 0.008, *r* = 0.335; (*n*_*PD*_ = 15; *n*_*con*_ = 19); MMGbra: *t*(24) = 2.079, *p* = 0.048, *r* = 0.39 (*n*_*PD*_ = 13; *n*_*con*_ = 13); MMGpect: *p* = 0.171; (*n*_*PD*_ = 12; *n*_*con*_ = 13). Thereby, the more affected side in PD patients with an UPDRS side difference of more than 2 pts indicated a rather lower MQ_REL_ compared to the less or not affected side (Fig. 5) (MMGbi: *t*(11) = 1.470, *p*_*MMGbi*_ = 0.170, *n*_*less*_ = 7, *n*_*more*_ = 6; *t*(6) = 0.884, *p*_*MMGbra*_ = 0.411, *n*_*less*_ = 4, *n*_*more*_ = 4; *t*(8) = 0.858, *p*_*MMGpect*_ = 0.416 , *n*_*less*_ = 5, *n*_*more*_ = 5). In those PD patients, the side asymmetries concerning the ratio MQ_REL_ in MMGbi were the highest with an amount of 19.4 ± 13.5 pp (range: 7.5 – 42.89) between the more and the less affected limb. The patients with a UPDRS difference of less or equal 2 points showed a relative side asymmetry of 13.7 ± 7.6 pp (range: 6.7 – 26.8). The relative side asymmetry of controls was found to be 8.4 ± 6.2 pp (range: 0.3 – 22.93) (Fig. 6).

The coefficient of variation of the Q_REL_ (CVQ_REL_) of the MMG-signals showed no significant result concerning MMGbi (*p* = 0.268; *n*_*PD*_ = 33; *n*_*con*_ = 39) and the MMGpect (*p* = 0.171, *n*_*PD*_ = 29; *n*_*con*_ = 32), respectively. The CVQ_REL_ was significant for the MMGbra in comparing the total sample of PD and Con (*U* = 601.00, *p* = 0.048; *r* = 0.25; *n*_*PD*_ = 29; *n*_*con*_ = 32), whereby the PD group showed higher CVs compared to the controls.

The CIs for the M and CV of the Q_REL_ of the ACC-signals were markedly overlapped in the unilateral trials and no significance was to be found (Fig. 3).

### Further parameters (3) to (5) of oscillatory signals

#### MMG-signals

**Figure 7:**
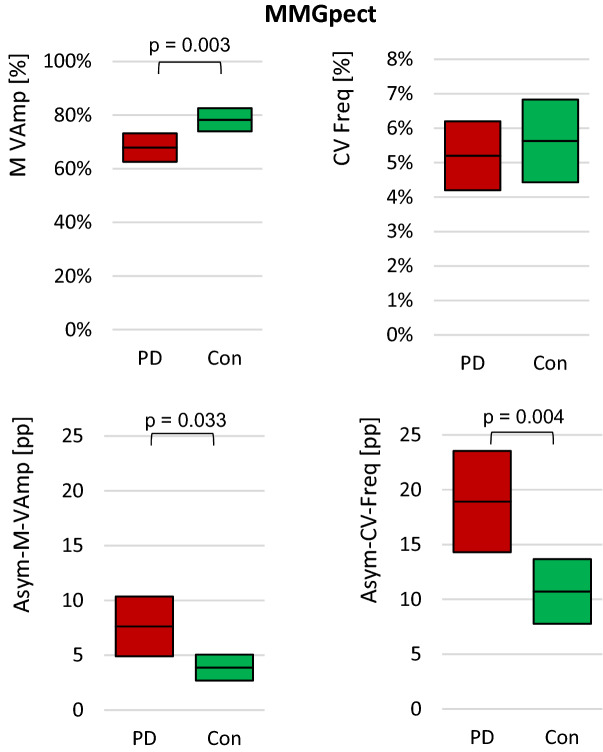
Oscillatory parameters of MMGpect. Displayed are the 95%-CIs of the parameters VAmp and frequency of the MMGpect (above) and of the related interlimb asymmetries (below). The M of VAmp differs significantly with *p* = 0.003, *r* = 0.34 and the CV of frequency shows no significant difference between the total group of PD and Con. The side differences, however, are significantly different with *p* = 0.033 (*r* = 0.37) for Asym-M-VAmp and *p* = 0.004 (*r* = 0.48) for Asym-CV-Freq.

The MMG-signals of biceps brachii and brachioradialis muscles showed no significant differences between PD and controls concerning mean frequency, amplitude variation and slope (*p* = 0.088 – 0.964) (Table 4). The arithmetic mean of VAmp of the MMGpect differed significantly between PD and Con (*t*(73) = 3.081, *p* = 0.003, *r* = 0.34, *n*_*PD*_ = 33, *n*_*Con*_ = 42) (Fig. 7, Table 4). The side asymmetry was found to be higher in the PD group compared to controls with an effect size of *r* = 0.37 (Asym-M-VAmp: *U* = 201.00, *p* = 0.033) (Table 4).

**Table 4:** Arithmetic means and standard deviations (M ± SD) of the oscillatory parameters (3) to (5) of the ACC and MMG signals including side asymmetries (Asym), significance *p* and effect size *r* of PD vs. Con.

	PD		Con		Sign. p	Effect r
	Less affected	More affected	left	right		
**ACC (n**_**PD**_** = 22; n**_**Con**_** = 26)**						
Slope [°]						
M	3.60 ± 3.10	4.76 ± 4.82	2.32 ± 4.69	1.71 ± 1.97	**0.022**	**0.33**
CV	1.25 ± 1.8	2.49 ± 5.35	3.41 ± 4.35	3.56 ± 3.02	**0.004**	**0.42**
Asym-M	4.12 ± 4.68		3.12 ± 2.95		1.00	-
Asym-CV	16.54 ± 17.14		19.39 ± 11.08		0.71	-
VAmp						
M	0.61 ± 0.17	0.54 ± 0.22	0.60 ± 0.23	0.57 ± 0.23	0.82	**–**
CV	0.21 ± 0.09	0.17 ± 0.09	0.20 ± 0.09	0.20 ± 0.09	0.79	–
Asym-M	8.14 ± 4.74		6.14 ± 6.61		0.14	-
Asym-CV	7.69 ± 5.47		15.03 ± 10.70		0.10	-
Frequency [Hz]						
M	9.96 ± 1.4	9.77 ± 1.4	9.93 ± 1.5	9.60 ± 1.2	0.87	–
CV	0.07 ± 0.03	0.06 ± 0.02	0.07 ± 0.03	0.07 ± 0.03	0.31	–
Asym-M	2.55 ± 1.65		2.01 ± 2.30		0.17	-
Asym-CV	9.67 ± 6.16		12.43 ± 8.69		0.76	-
**MMGbi (n**_**PD**_** = 37; n**_**Con**_** = 46)**						
Slope [°]						
M	0.07 ± 0.19	− 0.05 ± 0.18	0.14 ± 0.46	0.007 ± 0.01	0.833	–
CV	8.12 ± 18.55	2.67 ± 4.45	4.90 ± 7.21	3.85 ± 5.68	0.682	–
Asym-M	0.16 ± 0.11		0.30 ± 0.44		0.680	-
Asym-CV	22.87 ± 12.71		24.22 ± 16.21		0.953	-
VAmp						
M	0.81 ± 0.11	0.85 ± 0.08	0.83 ± 0.11	0.82 ± 0.13	0.832	**–**
CV	0.12 ± 0.04	0.11 ± 0.05	0.13 ± 0.06	0.15 ± 0.09	0.252	–
Asym-M	3.95 ± 2.58		2.84 ± 2.33		0.249	-
Asym-CV	12.63 ± 9.44		9.88 ± 8.62		0.366	-
Frequency [Hz]						
M	15.39 ± 1.5	15.05 ± 1.0	14.76 ± 1.5	14.22 ± 1.4	0.088	–
CV	0.037 ± 0.02	0.048 ± 0.02	0.05 ± 0.02	0.05 ± 0.04	0.216	–
Asym-M	2.36 ± 2.45		2.14 ± 1.29		0.964	-
Asym-CV	13.41 ± 8.52		13.94 ± 9.70		0.891	-
**MMGbra (n**_**PD**_** = 29; n**_**Con**_** = 32)**						
Slope [°]						
M	0.066 ± 0.27	− 0.039 ± 0.19	0.002 ± 0.26	− 0.092 ± 0.60	0.912	–
CV	3.62 ± 5.52	4.72 ± 6.79	4.31 ± 6.35	3.81 ± 5.10	0.923	–
Asym-M	0.21 ± 0.264		0.32 ± 0.498		0.545	-
Asym-CV	4.88 ± 6.76		5.86 ± 7.21		0.724	-
VAmp						
M	0.983 ± 0.20	0.898 ± 0.15	0.999 ± 0.17	0.968 ± 0.23	0.211	**–**
CV	0.14 ± 0.06	0.11 ± 0.05	0.09 ± 0.04	0.12 ± 0.08	0.335	–
Asym-M	3.638 ± 2.41		3.39 ± 2.87		0.511	-
Asym-CV	12.95 ± 11.16		9.51 ± 5.64		0.724	-
Frequency [Hz]						
M	15.58 ± 1.50	15.26 ± 1.65	15.65 ± 1.21	15.848 ± 1.64	0.383	–
CV	0.051 ± 0.038	0.049 ± 0.021	0.057 ± 0.02	0.043 ± 0.022	0.663	–
Asym-M	1.61 ± 1.52		2.31 ± 1.63		0.223	-
Asym-CV	11.72 ± 11.89		13.71 ± 10.39		0.447	-
**MMGpect (n**_**PD**_** = 33; n**_**Con**_** = 42)**						
Slope [°]						
M	0.15 ± 0.43	0.04 ± 0.34	0.06 ± 0.34	− 0.01 ± 0.22	0.848	–
CV	23.82 ± 72.2	2.74 ± 3.2	7.04 ± 20.3	5.58 ± 12.0	0.898	–
Asym-M	0.353 ± 0.501		0.246 ± 0.258		0.545	-
Asym-CV	25.15 ± 12.77		20.83 ± 16.69		0.457	-
VAmp						
M	0.67 ± 0.16	0.69 ± 0.16	0.77 ± 0.14	0.79 ± 0.14	**0.003**	**0.34**
CV	0.13 ± 0.05	0.13 ± 0.07	0.16 ± 0.07	0.15 ± 0.11	0.236	–
Asym-M	7.63 ± 5.21		**3.88 ± 2.69**		**0.033**	**0.37**
Asym-CV	11.51 ± 9.51		9.18 ± 8.16		0.457	-
Frequency [Hz]						
M	12.94 ± 1.15	13.11 ± 2.1	13.05 ± 1.1	13.68 ± 1.1	0.276	**–**
CV	0.05 ± 0.02	0.06 ± 0.04	0.06 ± 0.03	0.06 ± 0.05	0.781	–
Asym-M	3.37 ± 2.93		1.93 ± 1.59		0.120	-
Asym-CV	18.92 ± 8.83		**10.72 ± 6.74**		**0.004**	**0.48**

Significant results are displayed in bold.

The frequency of MMGpect varied slightly more in Con (n.s., *p* = 0.781), but the relative interlimb asymmetry of the CV of the frequency was significantly higher in PD with an effect size of *r* = 0.48 (Asym-CV-Freq: *t*(31) = -2.739, *p* = 0.004, *n*_*PD*_ = 14, *n*_*Con*_ = 20). (Fig. 7, Table 4).

#### ACC-signal

**Figure 8:**
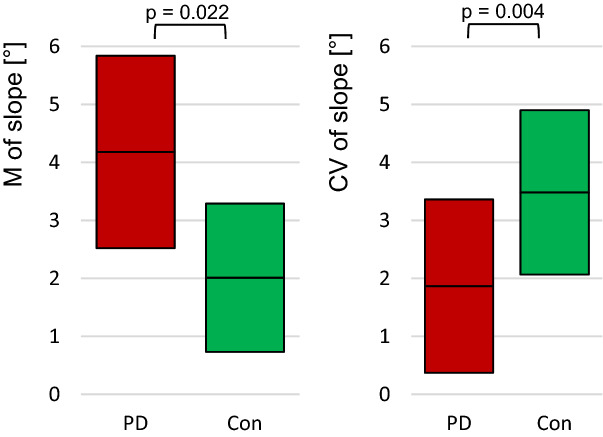
Slope of the amplitude maxima of ACC. Displayed are the 95% CIs of the arithmetic mean (left) and the coefficient of variation (right) of the slope of the amplitude maxima of the ACC signal. Both show significant results (M: *p* = 0.022, CV: *p* = 0.004; n_PD_ = 22, n_con_ = 26). The PD show higher mean slope than the controls, whereas the PD have less variation between the five trials.

The slope of amplitude maxima of the ACC-signal differed significantly between PD and Con with regard to the M and CV of the five trials. The M of slope of PD was significantly higher compared to the Con group (M slope: *U* = 2.297, *p* = 0.022, *r* = 0.33, *n* = 48), whereas the CV of slope was significantly lower in PD (*U* = -2.876, *p* = 0.004, *r* = 0.42) (Fig. 8). Significant interlimb asymmetries did not exist (Table 4).

#### UPDRS

The UPDRS scores for the healthy controls were 0 in all cases. In PD patients they ranged from 0 to 17 points for the right and from 0 to 11 points for the left side and showed no statistical differences between left and right side (*W* = 31.5, *p* = 0.103). The interlimb differences ranged from 0 to 8 points. Eight out of the 22 patients showed a difference of more than 2 points. Six patients exhibited severer symptoms in the neurological examination on the right side, two on the left side.

In comparing the UPDRS and the own assessment of patients with respect to the more affected side, nine cases indicated a different estimation. However, only in three of those patients the UPDRS difference between left and right side was higher than 2 points.

In the remaining five patients with an UPDRS difference higher than 2 pts, the own assessment and the estimation using the UPDRS agreed. The UPDRS score had no significant influence on all considered parameters (*p* > 0.05).

## Discussion

The objective of the study was to evaluate the oscillatory patterns of the armflexors and pectoralis muscle during a unilateral motor task in PD patients without tremor. The presented results show that the majority of comparisons are not to be found significant. This is not in accordance with the corresponding investigation using a bilateral motor task in the same participants^28^. However, the consideration of interlimb asymmetries seem to be relevant in the unilateral task: the PD patients showed higher side differences for the arithmetic mean of power ratio MQ_REL_ in MMGbi and MMGbra as well as for the arithmetic mean of amplitude variation and the CV of mean frequency of MMGpect. Furthermore, the ACC signal shows differing behaviour of slope of amplitude maxima, whereby the mean of slope is higher in PD, but the PD patients show lower variability between the trials (CV). Before presenting a content-related discussion of the results, the limitations have to be considered.

### Methodological limitations

#### Proportion of gender

The amount of female PD patients was clearly lower than the proportion of male PD patients (*n* = 4 vs. *n* = 17). This is justified by the clinical practice. Female Parkinson patients, who meet the inclusion criteria, were rare. With regard to the pilot character, we decided to accept this difference in gender proportion in the groups. Since MMG is gender-neutral^31^ and the investigated parameters did not differ statistically with respect to gender, the potential for error is assessed to be minor.

The force level is more relevant with regard to a probable influencing effect on the amplitude of the oscillatory signals. The force differences between the groups PD and Con were not to be found significant. Especially in male, however, the PD group had a lower MVIC compared to controls (left ≈ -11 Nm, *p* = 0.530; right ≈ -12 Nm, *p* = 0.270). Although the differences were not statistically relevant, this still might have influenced the signal quality. Since only relative parameters were considered, the outcome should not be influenced thereby.

#### Signal quality

Several signals had to be excluded due to the signal quality. According to Husar^29^ the SNR has to amount at least 10 dB for analysing the oscillatory behaviour of data. Therefore, all signals with a lower SNR were excluded. Due to the exclusions, the sample sizes are reduced for some comparisons (Table 2).

#### Multiple testing

Within this explorative study, a large amount of comparisons were performed (*n* = 64), because the results should be compared to the results of the measurements in a bilateral motor task, which were conducted in the same population. This was done to get insights into different characteristics of the muscular oscillations in this explorative study design^28^. Some authors stated that in explorative studies or non-clinical studies, a correction for multiple testing is not mandatory^32–34^. However, it has to be mentioned, that using the Simes procedure for multiple testing no comparison turned out to be significant anymore.

The setting of the unilateral motor task might have also influenced the results. One has to take into account that the oscillation pattern might be affected by the fixation of the force sensor on the plate of the chair. This might probably have dampened the oscillations.

The results have to be considered with caution in any way, due to the explorative character, the small sample sizes and the multiple comparisons. However, the results still reveal insights into probable mechanisms and characterisations of the mechanical muscle oscillations in PD patients without tremor.

### Content-related discussion

#### Power frequency distribution in patients with Parkinson’s disease

It was primarily hypothesized that the frequency of mechanical muscular oscillations would be shifted to lower frequency bands in PD patients due to the commonly appearing tremor of ~ 5 Hz in motor state^35^. It was assumed that this shift might be visible in the muscular micro-oscillations in PD patients without tremor, thus, prior to the manifestation of the Parkinsonion tremor. The presented results indicate that the shift seems to be diametrical. Considering the MMG signals, the arithmetic mean of power in I1 (3 to 7 Hz) amounts approximately 28 ± 6% of the entire frequency range of 3 to 12 Hz in controls, whereas in PD the power of I1 amounts to about 19 ± 10% (*p* = 0.001 – 0.225) of the total frequency range considered. This indicates that the power in the low-frequency range is relatively higher in controls. Thus, there is a different power proportion, however, not in the hypothesized way. This differing power ratio already was found for the bilateral motor task^28^. The changed pattern is also visible for the PD patients considering the less and more affected side, although this comparison is not significant (UPDRS difference > 2 pts.): I1 amounts approximately 27 ± 15% for the less affected side and 20 ± 11% for the more affected side (*n* = 8, *p* > 0.05). The mechanical muscle oscillations must be an expression of neuronal activity, which reflects the motor control^16^. Nevertheless, MMG only reflects the mechanical motor output. Thus, if at all, it can provide indirect insights into central processes. A lot of research has been performed regarding oscillations of neuronal structures in PD patients with tremor, e.g. of the subthalamic nucleus as part of the basal ganglia. Even if concrete details of the activity in the involved neuronal networks still remain widely unclear^36–38^, the beta band oscillations seem to play an important role in the pathophysiology of PD^39–42^. Probably, the changed power frequency pattern reflects those changes of central processes. A more detailed discussion concerning the central oscillations with respect to the mechanical muscular oscillations was provided in the article of the bilateral tasks^28^. It would be conceivable that the shift of the power frequency pattern to higher frequency ranges might reflect a counter regulation in PD patients prior to the manifestation of the tremor. Probably, the neuromuscular system up-regulates the frequency of motor control as a last opportunity to prevent the low-frequency tremor. Therefore, we suspect that this shifted pattern might probably be specific for PD patients without tremor.

### Changed variability in PD patients as a rather unspecific change of motor control

In several other neuromusculoskeletal diseases, changes in variability are detectable between healthy people and patients. A lower variability in patients was shown for gait-lines in herniated disc of lumbar spine^43^ or gait lines in patients with orthopedic complaints of the lower limbs^44^.

A higher variability in patients with Parkinson’s and Huntington’s disease was shown for parameters as stride duration^18,19,23^ as well as swing and step time^17,19–21^, stride length^45^ or other postural adjustements during gait^46^. Obviously, it is crucial, which parameter is examined. The parameters which are necessary to balance or to adapt adequately to external forces (balance during one step, amplitude variation during isometric muscle action, etc.) seem rather to decrease, whereas the variation of parameters of a specific motor programme (as swing and step time etc.) seem rather to increase.

In the presented study, the variation of the amplitude maxima of the MMGpect within one trial is lower in PD with 68 ± 16% compared to Con with 78 ± 14% (*p* = 0.003), whereby the interlimb asymmetry of this parameter is higher in PD (PD: Asym-VAmp = 7.6 ± 5.2 pp; Con: Asym-VAmp = 3.9 ± 2.7 pp; *p* = 0.033). The reason why this pattern is not apparent in the armflexors only can be assumed. Probably, the setting up also influences this parameter. It has to be taken into account, too, that the results might be random.

### Interlimb asymmetries might reflect the unilateral onset of motor symptoms in PD

The relative interlimb differences between left and right side appear to be the most recurring significant parameter in the unilateral measures. Concerning the mean of the ratio Q_REL_ of the MMG of armflexors as well as concerning the mean of amplitude variation and the CV of mean frequency of the MMGpect the side asymmetries differed significantly between PD and Con (*p* = 0.003 – 0.048). Thereby, the PD always showed a higher interlimb asymmetry. It is assumed that the side difference concerning neuromuscular parameters should be low in healthy persons as indicated for the controls in the presented study. This is supported by other investigations, in which non-significant side differences have occured in healthy controls concerning neuromuscular parameters as reflex latencies of the semitendinosus tendon^47^ or concerning the stride-to-stride variability^23^. While in diseases as Parkinson’s, the stride-to-stride variability^23^, the arm swing during walking^48^ or the balance control^49^ showed a higher interlimb asymmetry. Furthermore, PD patients exhibited deficits in interlimb coordination^25^, which is related to the interlimb asymmetry.

Especially the balance control seems to be closely related to the performed unilateral motor task in the presented study, since both tasks—balance control and controlling a given force intensity—refer to closed-loop systems. Both rely on proprioception and on feedback control to make adjustments during the tasks. The pertubations during the balance tasks of Boonstra et al.^49^ might have required higher demands on neuromuscular adjustments compared to the presented study. Thereby, the participants just had to control the given force level by pulling on a strip and using a visual feedback. However, we assume that the neuromuscular control processes in the close loop system might be affected in PD.

The interlimb asymmetry concerning the parameter MQ_REL_ in the MMG signals of armflexors might be a precursor of the later inserting tremor, which is characterised by a unilateral onset^10,50^. We hypothesise that the side differences of the ratio might be specific for PD, whereas the other oscillatory parameters are potentially reflecting a more general change in motor control and might also be present in other diseases of the neuromusculoskeletal system.

### Difference of limb accelerations and mechanical muscular micro-oscillations

The parameter slope of amplitude maxima is significant for the ACC signal, which reflects the changes of amplitude extents of limb accelerations within one trial. The mean of slope is higher in PD patients compared to healthy control, whereby the CV between the five trials is lower in PD. That indicates that the amplitudes are increasing in the course of one trial, but between the trials the slope is rather steady. An increasing amplitude is known for fatiguing trials. Probably, the task was more exhausting for the PD patients and, therefore, the slope was higher.

Taking the results of MMGs and ACC together, a relevant methodological conclusion is that the ACC showed different results compared to the MMG signals. This indicates that monitoring the mechanical muscular oscillations could provide different results than recording the accelerations of an extremity. Moreover, the MMGs seem to provide further insights into the motor control due to the above mentioned results.

### Classification of results concerning the UPDRS

The sample size was very small considering only patients with a side difference in UPDRS of more than 2 pts and in which the signals of both sides were suitable (MMGbi: *n* = 6, MMGbra: *n* = 3, MMGpect: *n* = 3). Therein the reason for the non-significant differences between the less and more affected limb might be found. However, the interlimb asymmetries concerning the ratio MQ_REL_ were the highest in the PD patients with an UPDRS side difference of more than 2 pts. with an amount of averagely 19.4 ± 13.5 pp (range: 7.5 – 42.89 pp) between the more and the less affected limb in MMGbi signals. In the patients with an UPDRS difference of less or equal 2 points the relative interlimb asymmetry of MQ_REL_ reduced to averagely 13.7 ± 7.6 pp (range: 6.7 – 26.8 pp). In controls this trend continued and the interlimb asymmetries reduced further to 8.4 ± 6.2 pp (range: 0.3 – 22.93 pp). However, the sample size is too small to draw conclusions from this result. Nevertheless, the estimation using the UPDRS and the measurements seem to still rather agree.

Another point, which has to be considered critically concerning the UPDRS, is the dissonance between the scores taken by the physicians and the assessment of the patients. However, this was the case in only three patients with a UPDRS side difference of 3, 5 and 6 pts, respectively. The other patients, in which the dissonance appeared, had an UPDRS side difference of equal or lower than 2 pts. Probably, the differences are so marginal that the own perception and the estimation by UPDRS might differ. Nevertheless, one would expect an accordance between perception and estimation using the UPDRS. The reason for the discordance cannot be determined appropriately here. There might be a misjudgment on both sides. The patients may have mixed up the sides. However, the main problem of the UPDRS is the lack of objectivity, even though in studies with exercised testers, the reliability of the UPDRS is stated to be reliable and valid^5,6^. In several other studies, it was postulated that the inter rater reliability is only acceptable^7–9^. Evers et al.^11^ conclude that there is the need of a more reliable instrument. Probably, the assessment using the oscillatory behaviour of muscles during an isometric uni or bilateral motor task might support the diagnosis in the future. Further investigations have to re-examine the found results on a larger group of PD patients. Additionally, the participant groups have to be extended to other neurodegenerative diseases to assess the specifity of the found results. Thereby, it is suggested to only measure the MMG of pectoralis major and biceps brachii muscle, since the signals of brachioradialis muscle did not reveal further information.

### Comparison of the bilateral and the unilateral motor task

Almost all investigated parameters showed a clearer difference between PD and Con during the bilateral task (recently presented in^28^) compared to the unilateral task presented here. In the bilateral task, the participant pushed with both hands against the measuring device including a strain gauge and accelerometer infront of its chest. Probably, the bilateral task enhances the potentially pathological patterns of motor control, which are reflected in the mechanical muscle oscillations. Especially, the power frequency ratio and the amplitude variation within one trial showed altered patterns in the bilateral task in PD patients. It would be conceivable that the neuromuscular adjustments in the close loop system are more demanding in the bilateral task, since both hemispheres have to mutually adjust to coordinate the action and reaction between both extremities. Since interlimb coordination seems to be reduced in PD patients, this result would be supported by other studies^46,48^. Therefore, the more complex bilateral task might be more vulnerable with respect to changes in motor control patterns.

The unilateral task probably could be more sensitive for side differences. The bilateral task might transfer the pathological changes to the other side. Hence, the side asymmetries are levelled in the bilateral task. This could indicate that the assumed pathological changes in motor control are dominant or, probably, are enhanced in a bilateral task, like known for the Jendrássik maneuver^51–53^.

For further studies, especially the bilateral task might be promising to indicate changed pattern in PD patients in general, whereby the unilateral task might be appropriate to indicate interlimb asymmetries. However, due to the explorative character of the study, it has to be taken into account, too, that the results might be an effect of multiple testing and, therefore, might be random.

## Conclusion and outlook

The need of a more objective diagnostic tool in PD, especially for early stages, is widely accepted. In the present study a potential novel approach was proposed. The novelties are, in particular, that PD patients *without tremor* were investigated in a loaded motor task. Usually patients with tremor are examined under unloaded conditions. However, the results can only be interpreted as first hints.

The evaluation concerning the wavelet coherence of the MMG and ACC signals between the left and right upper extremity as well as between the muscles of one side is remaining.

Further investigations firstly have to verify the results in healthy persons, patients with PD and with other neurodegenerative diseases. Secondly, it has to be investigated whether or not the changes are specific for PD or if they occur in other or even most neurodegenerative diseases. It might be possible that specific deviation patterns exist for different diseases. Probably, several parameters of motor control as the power-frequency-ratio, the amplitude variation and the interlimb differences during loaded motor tasks might be collectively considered to support the diagnostics of PD in the future.

A development of a neuromuscular biomarker could have a large and essential impact on the diagnosis and follow-up in Parkinson’s disease. Moreover, the understanding of the underlying pathomechanism might be further investigated by implementing similar uni and bilateral motor tasks.
